# HIV and Vertebral Fractures: a Systematic Review and Metanalysis

**DOI:** 10.1038/s41598-018-26312-9

**Published:** 2018-05-18

**Authors:** Thales A. S. H. Ilha, Fabio V. Comim, Rafaela M. Copes, Juliet E. Compston, Melissa O. Premaor

**Affiliations:** 1 Pós-graduação em Farmacologia, Health Sciences Center, Federal University of Santa Maria, Santa Maria, Brazil; 2 Department of Clinical Medicine, Health Sciences Center, Federal University of Santa Maria, Santa Maria, Brazil; 3 Cambridge Biomedical Campus, Cambridge, United Kingdom

## Abstract

The survival of HIV-infected patients has increased with the advent of antiretroviral therapy with the emergence of new comorbidities. Vertebral fracture is a manifestation of reduced bone strength and osteoporosis. This study aims to assess the frequency of spine fractures in HIV-positive men and women aged over 18 years. We performed a systematic review of randomized controlled trials, cohort studies, cross-sectional studies, and case-control studies. Studies that evaluated morphometric and/or clinical vertebral fracture were included. In total 488 studies were found, of which 53 had their full texts evaluated. A total of 85,411 HIV positive individuals were identified in 26 studies. The meta-analysis of the prevalence of vertebral fractures included 12 studies with 10,593 subjects. The prevalence was 11.1% [95% confidence interval (95% CI) 4.5%, 25.0%, I^2^ 98.2% p < 0.00001]. When we evaluated independently studies of clinical vertebral fracture and morphometric vertebral fracture, the prevalence was 3.9% (95% CI 0.9, 15.8, I^2^ 96.4% p < 0.00001) and 20.2% (95% CI 15.7%, 25.6%, I^2^ 69.9% p = 0.003) respectively. HIV-infected individuals had an odds ratio of vertebral fractures of 2.3 (95% CI 1.37, 3.85, I^2^ 98.2% p < 0.00001) when compared with HIV-uninfected patients (n = 9 studies). In conclusion, HIV-positive subjects had a higher risk of vertebral fractures when compared with HIV-negative subjects.

## Introduction

With the advent of antiretroviral therapy (ARV), the survival of HIV-positive individuals has increased^1,2^. Consequently, the spectrum of comorbidities they exhibit has increased^3^. Many of these comorbidities, common in the older population, arise at a younger age in individuals infected with HIV and one of the affected systems is the skeleton. Although studies showing the incidence and prevalence of fractures in HIV-infected patients have different designs concerning the sample size, age and gender, study population, type of fracture, and method of fracture assessment, the vast majority show a significant increase in the risk of fracture^4^. Reduction in bone mineral density (BMD) has been reported in HIV-infected patients regardless of sex and age, with an odds ratio of 6.4 for reduced BMD and 3.7 for osteoporosis when compared with uninfected controls^5^.

Vertebral fractures are one of the most common manifestations of osteoporosis. Radiographically confirmed vertebral fractures are a sign of impaired bone quality and strength and a strong predictor of future vertebral and non-vertebral fractures^6^. The presence of one vertebral fracture is associated with a five-fold increase in the risk of subsequent vertebral fractures and a three-fold increase in hip fracture risk^7^. Most vertebral fractures are asymptomatic, and only about one-fourth of incident radiographic vertebral deformities are clinically diagnosed^8^. The prevalence of vertebral fractures in the general population increases with age, occurring in approximately 25% of women over 50 years^9^.

Recently, studies have demonstrated an increased prevalence of vertebral fracture in HIV patients^10–31^. Although the studies that evaluate the risk of vertebral fractures in people living with HIV appear to be consistent, the studies that evaluate the prevalence and incidence of spine fractures have shown different results. Moreover, these studies have different designs, sample sizes, and fracture assessment, which make the findings of each study difficult to interpret and generalize. Thus, we have performed a systematic review and meta-analysis to quantify the prevalence, incidence, and risk of vertebral fractures in HIV-individuals. To the best of your knowledge, this is the first metanalysis evaluating spine fractures in people living with HIV.

## Materials and Methods

The meta-analysis was carried out according to the PRISMA Guidelines^32^. The study was registered at PROSPERO, an international database of prospectively registered systematic reviews, with protocol number CRD42016048702 9 https://www.crd.york.ac.uk/PROSPERO/display_record.asp?ID=CRD42016048702). It was approved by the Research Committee of Health Sciences Centre of the Federal University of Santa Maria (045911).

Studies were included in the meta-analysis if they met the following criteria: (1) Randomized controlled trials, cohort studies, cross-sectional, and case-control; (2) men and women aged over 18 years, HIV-positive with or without antiretroviral therapy; (3) have evaluated both morphometric or clinical vertebral fracture; and (4) the primary outcome of interest was prevalence or incidence of vertebral fractures, and/or the secondary outcome was hazard ratio of fracture. Animal studies, studies that evaluated specific cohorts of patients with HIV (for example, studies that assess a particular variable only in patients e.g. HIV with hepatitis-C, or just HIV with lipodystrophy), or those that did not meet the inclusion criteria were excluded from the initial review. The last search was on September 27th, 2017.

The search for studies was performed in EMBASE (Elsevier), PubMed, the Regional Library of Medicine (BIREME), and the Cochrane Library (Cochrane Database of Systematic Reviews - CDSR). Also, studies based on the reference lists of the included articles were analyzed. Studies written in any language and with no publication date limits were considered. The terms used for the search are described in the supplementary material.

Whenever different articles from the same database were obtained, all the articles necessaries to complete the extraction were included in the metanalysis. The authors were contacted by email when more data were required.

### Selection process

The selection of the studies was performed by two protocol members independently. Firstly, the studies were screened based on their titles and abstracts. The studies that could not be ruled out in this procedure had their full texts evaluated. Additionally, for all selected items, the full texts were sought, and their eligibility was double-checked. If there was dis- agreement between the two reviewers regarding the identification, eligibility, and inclusion of items, they were checked again by a third reviewer and, if necessary by a fourth reviewer.

### Data collection process

The data of each study were extracted, independently, by two protocol members (TSI and RMC). The agreement between the two extractors should be 100%. In the cases where there was disagreement, a third and, if necessary, a fourth party adjudicated. The following data were extracted from each article: the name of the first author, year of publication, study design, site, age, gender, ART use, number of vertebral fractures, method of vertebral fracture evaluation (clinical, morphometric, ICD, self-reported), and outcome (vertebral fracture prevalence, incidence, odds/hazard ratio). When any of these data could not be extracted from the full text of the study, the authors were contacted.

### Risk of bias (quality) assessment

Assessment of the risk of bias of the included studies was performed independently by two authors and was ranked as high, low and uncertain. Possible discrepancies were adjudicated by the other protocol members. All included studies were cohort, randomized clinical trials, case–control, or cross-sectional studies. For RCTs and cohort or case-control studies, bias risk assessment was conducted by the Cochrane Collaboration tool^33^ and Newcastle-Ottawa scale^34^ respectively. The Newcastle-Ottawa scale assessed the selection, comparability and exposure of a case-control study and selection, comparability, and outcome of a cohort study. In it, 9 stars represent maximum score for a study, and the study with over 6 stars would be regarded as relatively high quality. For cross-sectional study assessment quality Crombie´s Scale^33^ was used. Crombie´s Scale is composed of 7 items, and each item is graded as “Yes” (1 point), “Unclear” (0.5 points), or “No” (0 points).

### Vertebral fracture definition

There are differing criteria for the definition of vertebral fracture and thus rates may vary within the same population depending on the approach selected. In addition, some studies use just ICD coding to detect the incidence or prevalence of vertebral fracture, which restricts data to inclusion only of fractures that present clinically. Because of the heterogeneity of methods used for vertebral fracture assessment among the studies, for the purposes of this analysis we separated the studies into two groups according to the approach utilised. Studies that used imaging methods (X-ray, DXA or CT), independent of protocol, were allocated to the morphometric vertebral fracture group. Studies that used ICD, self-report, or did not describe the method of vertebral fracture assessment were allocated to the clinical vertebral fracture group.

### Data synthesis and statistical analysis

Data on the prevalence, incidence, and the odds ratio of vertebral fracture were summarized separately. We pooled proportions (data for prevalence or incidence) applying LOGIT transformation, using random effects model, with DerSimonian and Laird as variance estimator^35^. Results were presented as a pooled proportion (prevalence or incidence), with 95% confidence intervals. The pooled odds ratio was estimated, also using random effects model, with DerSimonian and Laird as variance estimator^35^. The statistical heterogeneity among studies was assessed using Cochran’s Q test and the inconsistency I^2^ test^36^. We performed an additional analysis with a fixed-effects model to qualitatively evaluate differences in point estimates provided by models with random and fixed effects. For sensitivity analysis, to explain heterogeneity and potential effects modifiers, we performed subgroup analysis for the type of fracture assessment and type of study, and meta-regression for age, gender, study location, sample size, study year, and study quality using a mixed-effects model (with the explaining variable as fixed)^37,38^. The publication bias was evaluated thru a qualitative inspection of funnel plot^39^ and the Begg test as a statistical parameter for testing funnel plot asymmetry^40^. All the analyses were made using the software R [R version 3.2.4, 2016, The R Foundation for Statistical Computing, Platform: x86_64-apple-darwin13.4.0 (64-bit)] and RStudio [RStudio Team (2015). RStudio: Integrated Development for R. RStudio, Inc., Boston, MA http://www.rstudio.com/].

### Approval, Registration and Availability

Approval for this study was obtained from the Research Committee of Health Sciences Centre of the Federal University of Santa Maria (045911). The study protocol is registered with the International Prospective Register of Systematic Reviews (PROSPERO) under the number CRD42016048702. All research data will be available upon publication at https://www.dcmufsm.com.

## Results

### Study selection

**Figure 1:**
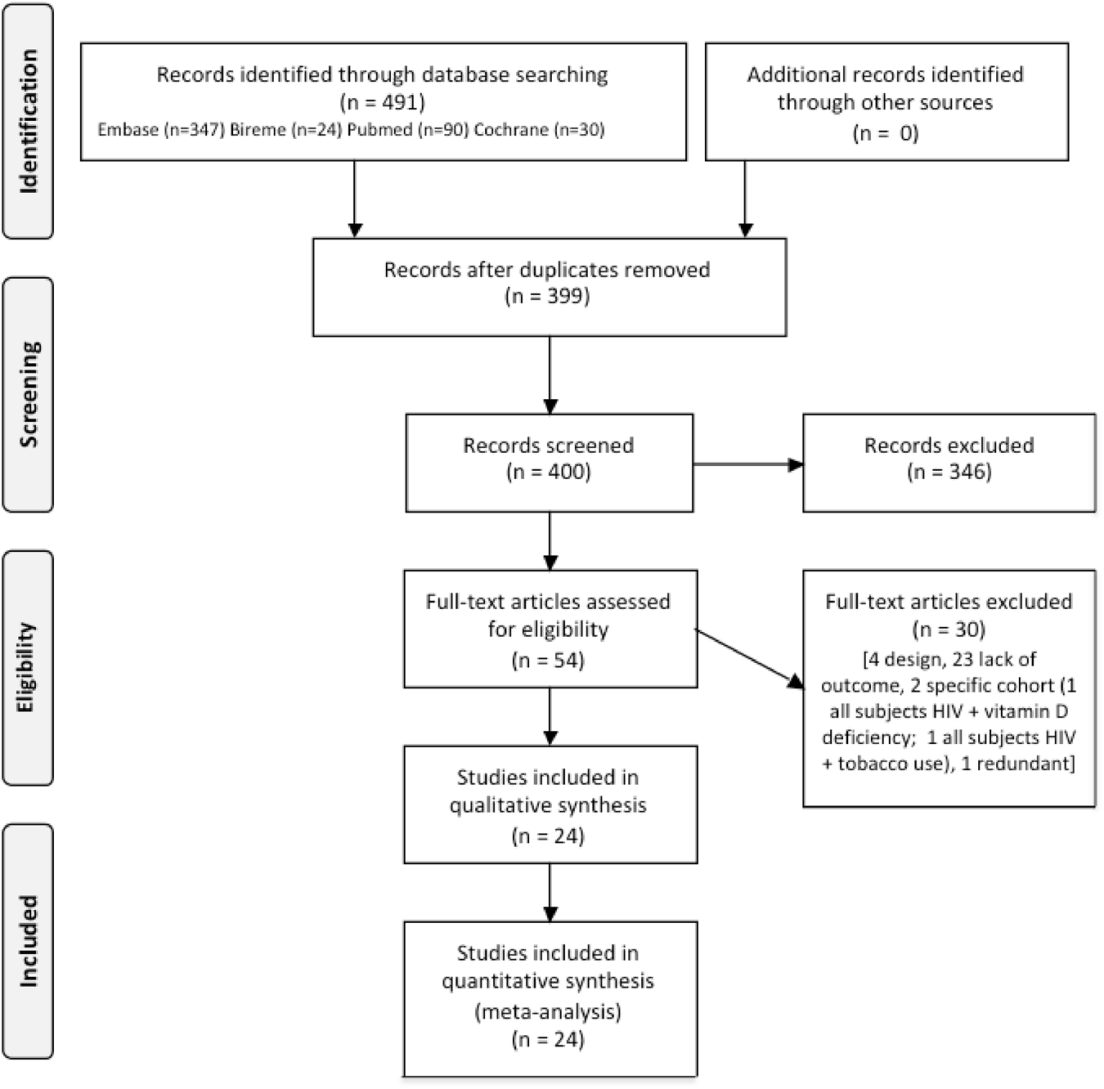
PRISMA 2009 Flow Diagram of the studies included in the review. The meta-analysis was carried out according to the PRISMA Guidelines^32^.

In total 488 studies were found in the electronic database search, of which 90 were duplicates. After screening the title and abstracts, 53 relevant studies remained and underwent detailed full-text review. Of these, 29 were excluded due to lack of a suitable outcome, inappropriate study design, inclusion of selected cohorts of patients with HIV, or redundant publication. A total of 24 articles met the eligibility criteria and were included in the meta-analysis. The process of relevant studies selection and the number of articles excluded at each stage are outlined in the Preferred Reporting Items for Systematic Reviews and Meta-Analyses (PRISMA) flow diagram (Fig. 1). In two different studies from the same author, carried out with the same cohort, the results were overlapping^25,26^. To achieve full information from this population, data were extracted from both articles and the values were pooled and analyzed together.

### Study characteristics

Table 1 describes the study characteristics of the 24 studies included in our meta-analysis. A total of 84,628 individuals HIV positive were identified in 24 studies. We found 10 cross-sectional studies with a total of 1,221 subjects; 10 cohort studies with 74,116; 3 case-control studies with 8,689 and 1 randomized clinical trial with 602. For vertebral fracture ascertainment, 9 studies used imaging methods, 7 used ICDs or databases, 4 used self-reported questionnaires, 1 used clinical charts, and 2 did not describe the method used. Seventeen studies recruited male and female participants, 5 studies recruited only males, and 2 studies recruited only females. Most of the subjects in the studies were taking antiretroviral therapy. Apart from the study conducted by Gallant *et al*.^17^, which was a multicenter study enrolling participants from several countries (South America, Europe, USA), the studies took place in individual countries: 8 from Italy, 7 from USA, 2 from Denmark and one each from Taiwan, France, Australia, UK and Japan.

**Table 1:** Studies characteristics.

Author	Year	Study design	Site	Age (years)	Gender (%)	Recruitment time (years of follow-up)	ART(%)	VF ascertainment
Bedimo^12^	2012	Cohort	USA	18–70+	98% male	1988–2009 (21)	69.4%	ICD
Borderi^13^	2014	Cross-sectional	Italy	51 (31–67)	68% male	NA	86.13%	Semiquantitative/Morphometric
Ciullin^14^	2017	Cross-sectional	Italy	43 (37–52)	87.2% male	NA	93.6%	Semiquantitative/Morphometric
Clò^15^	2015	Cross-sectional	Italy	47 (24–72)	64.15% male	NA	—	Lateral spine x-ray
Collin^16^	2009	Cohort	France	36.2	77.2% male	1997–2009 (7.1)	100%	—
Gallant^17^	2004	RCT	South America, Europe, USA	36	73.92% male	48 weeks	100%	Lateral spine x-ray
Gazzola^10^	2015	Cross-sectional	Italy	49 (40–51)	73%male	NA	70.61%	Semiquantitative/Morphometric
Hansen^18^	2012	Cohort	Denmark	36.7 (30.5–44.5)	76% male	1995–2009 (14)	78%	ICD
Kurita^19^	2014	Cohort	Japan	15–81	92.8% male	2005–2010 (5)	65,9%	—
Mazzotta^20^	2015	Cross-sectional	Italy	44.2 ± 10	70.6% male	NA	79.7%	Self-reported
Pepe^49^	2012	Cross-sectional	Italy	48.6 (40–69)	100% male	NA	100%	Semiquantitative
Porcelli^11^	2014	Cross-sectional	Italy	51 (36–75)	71% male	NA	100%	Semiquantitative/Morphometric
Prieto-Alhambra^21^	2014	Case-control	Denmark	43.4 ± 27.4	51.8% female	2009 (1)	—	ICD
Sharma^22^	2015	Cohort	USA	40 (34–46)	100% female	2002–2013 (10)	63%	Self-reported
Short^50^	2014	Cross-sectional	UK	45 (38–51)	100% male	NA	78%	Self-reported
Torti^23^	2012	Cross-sectional	Italy	53 (42–71)	100% male	NA	78.12%	Semiquantitative/Morphometric
Triant^24^	2008	Case- control	USA	20–79	65.16% male	1996–2008	—	ICD
Womack^*,^^25,26^	2011/2013	Cohort	USA	53 (48–61)	100% male	1997–2009 (6 ± 3.9)	75%	ICD
Yang^27^	2012	Cohort	Taiwan	<20–>60	76.9–90.1% male	2005–2008	—	ICD
Yin^28^	2012	Cohort	USA	39 (33–45)	83% male	(5)	99.69%	Self-reported
Yin^29^	2010	Cross-sectional	USA	55.9 ± 0.7	100% female	2002–2007	79.34%	Semiquantitative
Yong^30^	2011	Case-control	Australia	49.8	88.54% male	1998–2009 (10.5)	80.32%	ICD/Victorian HIV Database
Young^31^	2011	Cohort	USA	40 (34–46)	79% male	2000–2008	72.7%	Self-reported /HOPS electronic databases

—=information not given; NA = not assessed; RCT = Randomized Clinical Trial; ICD = International Code Diseases; ^*^The data were extracted from two different articles with the same cohort.

### Study quality

The Newcastle-Ottawa Scale was applied to assess the selection, comparability, and exposure of the case-control and cross-sectional study, and the selection, comparability, and outcome of the cohort study. These scores are displayed in Table 4 (Supplementary material). The Crombie’s items assessment for cross-sectional studies is described in Table 5 (Supplementary material).

### HIV and vertebral fracture

**Figure 2:**
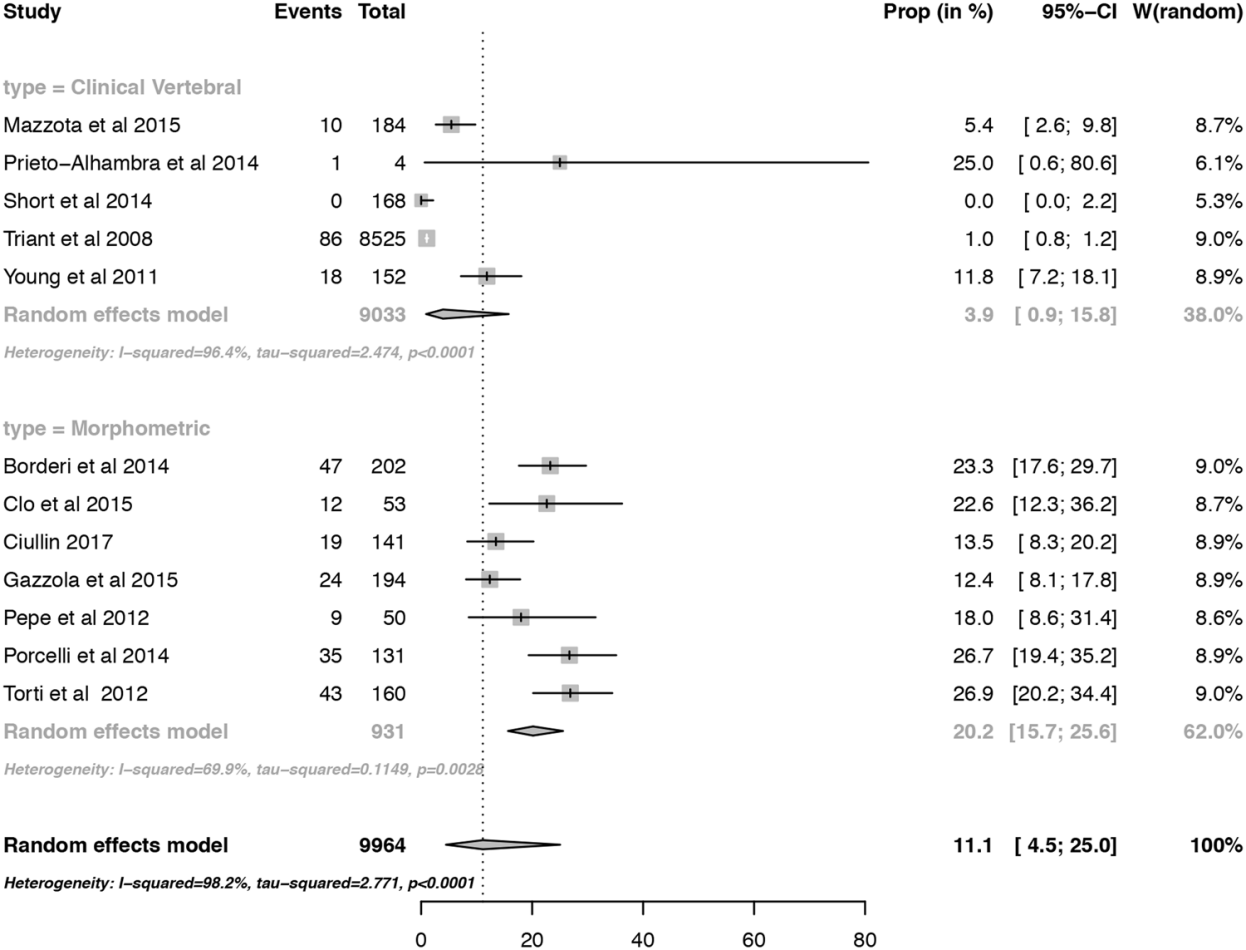
Forest plot of the prevalence of vertebral fractures in HIV-positive subjects. The proportions were pooled applying LOGIT transformation, using random effects model, with DerSimonian and Laird as variance estimator. Results were presented as a pooled prevalence, 95% confidence intervals. The p-value for subgroup analysis on the differences of the prevalence by type of fracture assessment is 0.021.

Twelve studies were used in the assessment of the prevalence of vertebral fracture in HIV-positive subjects (Fig. 2). The mean age of the included subjects varied from 40 to 53 years. Two subgroups were evaluated independently: clinical vertebral fracture and morphometric vertebral fracture, with 5 and 7 studies, respectively. The overall prevalence was 11.1% (95% confidence interval 4.5% to 25.0%; prediction interval 0.3% to 85.6%). The prevalence of morphometric vertebral fracture was 20.2% (95% confidence interval 15.7 to 25.6; prediction interval 8.8% to 39.6%), and of clinical vertebral fracture 3.9% (95% confidence interval 0.9 to 15.8; prediction interval 0.02% to 91.6%). The p-value for the publication bias evaluated by the Begg test was 0.131. The funnel plot is displayed in Figure 4, supplementary material. There were no major differences in the point estimates provided by models with random and fixed effects (data not shown).

A sensitivity analysis was carried out to investigate the effect of each study on results of the meta-analysis of the prevalence of vertebral fracture. We excluded the included articles one by one; however, the pooled results were not affected by this procedure and no single article could explain the source of the heterogeneity.

The meta-analysis of the incidence of vertebral fractures included 10 studies with 422,799 PY HIV- infected individuals. The pooled estimate of the incidence was 0.8 (95% confidence interval 0.4 to 1.9) per 1000 person-years. The assessment for heterogeneity was significant for vertebral fractures (*Q* = *1*.*459*, *p* = *0*.*0001*, *I*² = 95.9%). However, when we excluded one outlier study (Young *et al*. 2011), the incidence estimate decreased to 0.6 (95% confidence interval 0.4 to 0.8) per 1000 person-years and the heterogeneity remained significant, but lower (*Q* = *0*.*194*, *p* = *0*.*0001*, *I*² = 74.9%). The incidence rate of each study is described in Table 2.

**Table 2:** Details of Incidence Vertebral Fracture by Cohort.

Cohort	Persons/Years	Incident Vertebral Fracture^*^	95% - CI
Bedimo *et al*.^12^	305237	0.4	0.3–0.5
Collin *et al*.^16^	6380	0.5	0.1–1.4
Gallant *et al*.^17^	1533	0.7	0.0–3.6
Hansen *et al*.^18^	29348	0.6	0.4–1.0
Kurita *et al*.^19^	2805	0.7	0.1–2.6
Sharma *et al*.^22^	22520	1.2	0.8–1.7
Yang *et al*.^27^	12898	0.5	0.2–1.0
Yin *et al*.^28^	23200	0.3	0.1–0.6
Yong *et al*.^30^	25452	0.7	0.4 -. 1.1
Young *et al*.^31^	1216	14.8	8.8–23.3
**Total**	**430589**	**0**.**8**	**0**.**4–1**.**8**

^*^Per 1.000 persons/years; CI = confidence interval.

**Figure 3:**
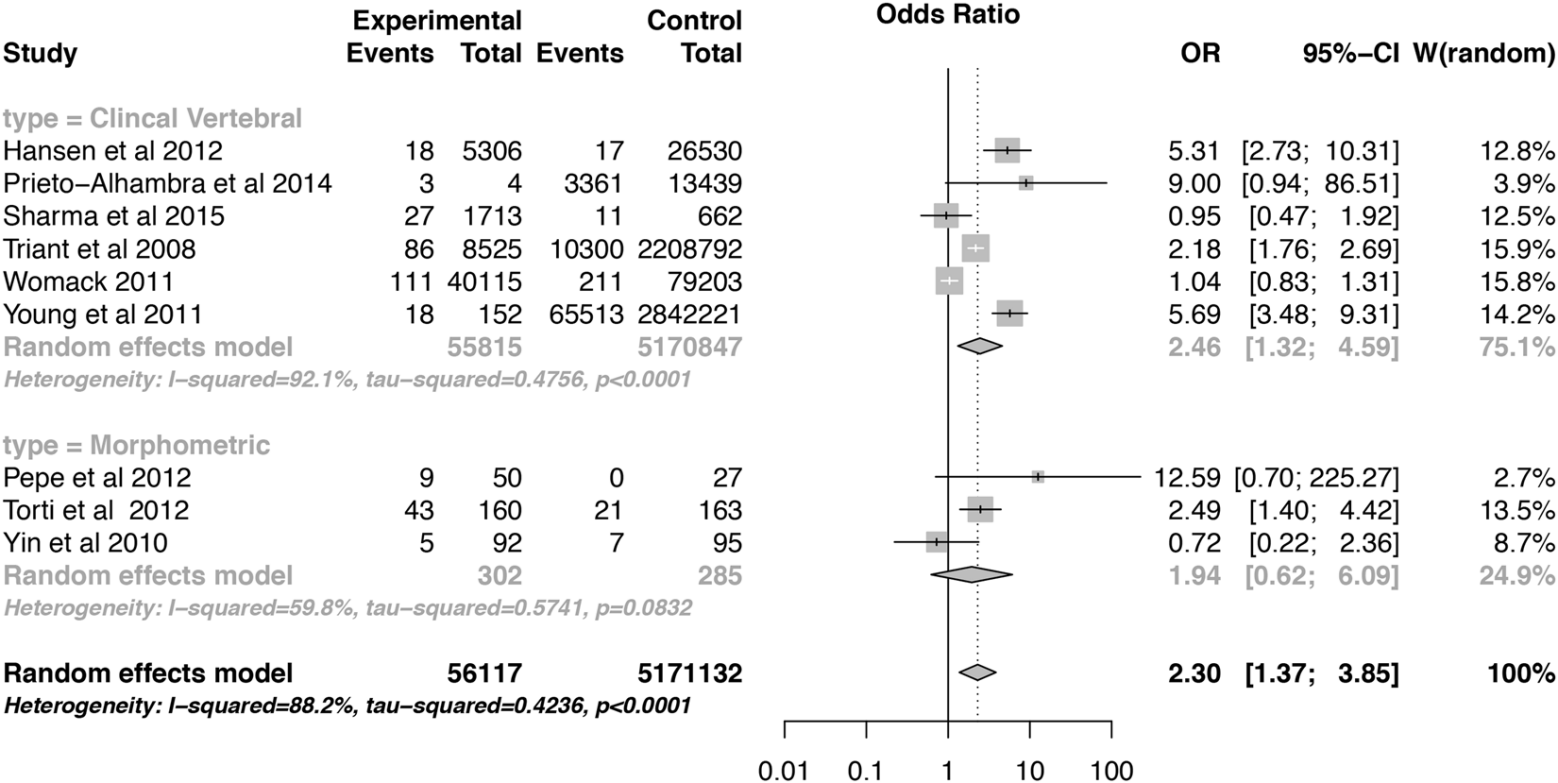
Forest plot of the odds ratio of vertebral fractures in HIV-positive subjects. The pooled odds ratio was estimated using random effects model, with DerSimonian and Laird as variance estimator. Results were presented as a pooled odds ratio, 95% confidence intervals. The p-value for subgroup analysis on the differences of the prevalence by type of fracture assessment is 0.211.

In a total of 9 studies, 56,117 HIV-infected patients were compared with 517,1132 HIV-uninfected controls. HIV patients had a 2.3-fold increase in the odds ratio of vertebral fracture (95% confidence interval 1.37 to 3.85) when compared with HIV-uninfected patients (Fig. 3). There was no interaction between the assessment method [morphometric or clinical vertebral, P = 0.211] and the study type (case-control and cross-sectional, P = 0.918]; (data not shown). The p-value for the publication bias evaluated by the Begg test was 0.421. The funnel plot is displayed in Figure 4, supplementary material. There were no major differences in the point estimates provided by models with random and fixed effects (data not shown).

### Meta-regression

The results for the meta-regression of the prevalence analysis are shown in Table 3. There was no interaction between age, gender, and study year and the prevalence of vertebral fractures. Nonetheless, the heterogeneity was explained, at least in part, by the study location, sample size, and study quality.

**Table 3:** Meta-regression analysis of the prevalence rate covariates.

	Subgroups	No of studies	RP	Summary effects (95% CI)	p Value	Heterogeneity	
						I², %	p Value
Meta-regression for age	Age (years)	12	0.07	−0.18, 0.34	0.553	98.31	<0.0001
Meta-regression for gender	Male	12	−0.01	−0.06, 0.05	0.846	97.70	<0.0001
Meta-regression for study location	Italy^*^	12	−1.02	−1.36, −0.67	<0.0001	79.22	<0.0001
Meta-regression for sample size	n	12	−0.0003	−0.0005, −0.0002	<0.0001	82.06	<0.0001
Meta-regression for study year	Year	12	0.22	−0.06, 0.50	0.192	95.06	<0.0001
Meta-regression for study quality	Moderate quality^**^	12	−1.74	−2.95, −0.54	0.0045	92.81	<0.0001

Main results of the meta-regression. C-C = case control studies; C-S = cross-section studies; VF = vertebral fractures; RP = prevalence ratio;

^*^when compared with UK; ^**^when compared with high quality.

## Discussion

This systematic review included 24 studies, with 84,628 HIV-infected subjects. The pooled results indicated that HIV infection was associated with an increase in the risk of vertebral fracture. When compared with HIV-negative subjects, HIV patients had a 2.3-fold increased risk of vertebral fracture, independent of age and gender.

Previous studies have shown that HIV infection has adverse effects on bone health. In 2006, a meta-analysis of cross-sectional studies reported an odds ratio of 6.4 for reduced BMD and 3.7 for osteoporosis, defined as a BMDT-score ≤−2.5 in HIV-infected subjects compared with uninfected controls^5^. In another meta-analysis, Shiau *et al*.^41^ found a modest increase in the incidence of fracture in HIV-infected individuals, with an incidence rate ratio of 1.35 (95% CI 1.10–1.65) for fragility fracture and 1.58 (95% CI 1.25–2.00] for any fracture.

The exact mechanisms underlying bone disease in the HIV-infected population remain unclear. Risk factors for fracture include many of those seen in the general population, for example smoking, alcohol abuse, and glucocorticoid therapy, as well as HIV-specific risk factors such as anti-retroviral therapy, co-infection with hepatitis C, and hypogonadism. In addition, co-morbidities associated with HIV infection such as renal disease and diabetes also increase fracture risk^42^.

In our study, the meta-regression did not show an association between age or gender and vertebral fracture. Although individual studies have included older subjects, the age range in the analyzed studies in our meta-regression was from 40 to 53 years, which may have contributed to this finding. The positive association between age and vertebral fracture prevalence in the general population has been well documented in several large population-based studies^43^. The narrow age range in our study could have blunted this association.

The influence of gender on vertebral fractures in the general population appears to vary with age. Studies that include men and woman have reported that the incidence is higher in men than women under the age of 50–55 years^43^. For men and woman aged 50–79 years, data from European Vertebral Osteoporosis Study (EVOS)^44^ have shown that the prevalence of vertebral fracture in the age-standardized population in Europe was 12.2% and 12% respectively. Other studies show that the risk rises in women after the age of 60 years and substantially after 70 years of age^45^. There was no interaction between gender and vertebral fracture in our study.

Although the vast majority of the subjects in the studies evaluated in this systematic review and meta-analysis were on ART, the type and the duration of therapy were not described in several studies; therefore, we could not study its influence on vertebral fractures. The effects of ART on bone mineral density in HIV infected individuals are well documented, with losses of between 2% and 6% during the first year of therapy and a tendency to stabilise thereafter, this effect varying with the drugs used. Bolland *et al*.^46^ investigated temporal changes in BMD of adults with HIV in longitudinal studies. The authors found that BMD was stable in HIV cohorts established on antiretroviral therapy, whereas cohorts initiating therapy had short-term accelerated BMD loss followed by a longer period of stability. To examine whether ART initiation is associated with increased fracture rate, Yin^28^ evaluated the incidence of fracture in HIV-positive subjects from 26 randomized ART studies (n = 4640) followed in the AIDS Clinical Trials Group (ACTG) Longitudinal-Linked Randomized Trial study for 5 years. The authors found that in ART-naive subjects, fracture rates were higher in the first 2 years after ART initiation [(0.53/100 person-years) when compared to the subsequent years (0.30/100 person-years)]. Nonetheless, the data on vertebral fracture incidence after initiation ART are sparse. In addition, most studies did not include morphometric evaluation of vertebral fractures.

The disparity between the prevalence of morphometric (20.2%) and clinical vertebral fractures (3.9%) in our study is in agreement with findings in the general population. Clinical vertebral fracture rates are considerably lower than radiographic vertebral fracture rates, reflecting the minority of these fractures that come to clinical attention^47^. In the general population, the prevalence of morphometric vertebral fractures in people age 50 years and over varies between 9.5 and 37%^43,47^, the value increasing with age and also varying according to the definition of vertebral fracture. In the EVOS study, the prevalence varied from 12.2% for men and 12% for women using the McCloskey method to 20.2% and 20.2%, respectively, when the Eastell method was used. In our analysis, studies using semiquantitative and quantitative methods were pooled together. This approach might have generated a conservative bias, underestimating the frequency of fractures. Studies that used the semiquantitative method could have missed mild fractures, classified as grade 1 by Genant^48^ because of the difficulty of detecting this type of fracture in younger individuals.

In our study, the data for vertebral fracture incidence were obtained from 10 studies. Nine of them used ICD data or self-reporting. Since only around one third of vertebral fractures are symptomatic, use of self-reporting data will underestimate the incidence. Furthermore different coding is used in ICD9 and ICD10, which may have contributed to the variation in reported incidence. The most recent version of ICD 10 defines several subtypes of vertebral fractures, and depending on the coding used by each study, different types and sites of vertebral fracture may be included. Hence, high impact fractures or fractures in cervical vertebrae may be included. Furthermore, the incidence ratio found in our study should be interpreted with caution as there were insufficient data to provide a robust estimate.

Our study has some limitations. It is a systematic review and meta-analysis of observational studies that aimed to evaluate the frequency and risk of vertebral fractures, therefore it is to be expected that our pooled analysis showed heterogeneity. Differences in the design of the studies, the study location, the sample size, the populations studied, the method used for vertebral fracture evaluation, and the quality of the studies may all have contributed to the observed heterogeneity. Another limitation is the fact that we included cohort, case-control, and cross-sectional studies in the estimated odds ratio, thus causality may not be concluded. Nevertheless, there was no interaction between the study type or method used for fracture and the pooled odds ratio of spine fractures. Finally, some studies included in our systematic review were not specifically designed to evaluate vertebral fracture and/or vertebral fracture was not the primary endpoint. Because many vertebral fractures are asymptomatic, they might be underreported in those studies. Hence the prevalence of clinical vertebral fractures and the incidence of vertebral fractures may be underestimated in our metanalysis.

Our study demonstrates that the risk of vertebral fracture in HIV-positive subjects is approximately double that of HIV-negative subjects, a finding that has important clinical implications. Although the risk factors for vertebral fractures have not been clearly established in the ‘HIV infected population, identification and correction of risk factors recognized in other populations, such as smoking, alcohol abuse, sedentary lifestyle, vitamin D deficiency, and the use of glucocorticoids should be recommended. Moreover, screening for spine fractures with X-ray should be considered in high risk individuals. Further studies are required to establish vertebral fracture incidence in HIV-positive individuals, to identify the risk factors involved, and to develop effective strategies for their prevention

## Electronic supplementary material

SUPPLEMENTARY MATERIAL
