# Supplementary material for: HIV and Vertebral Fractures: a Systematic Review and Metanalysis
Source: Sci Rep. 2018 May 18;8:7838. doi: 10.1038/s41598-018-26312-9 (PMC5959850; doi:10.1038/s41598-018-26312-9)
Supplement: Supplementary file 1 — SUPPLEMENTARY MATERIAL [file 41598_2018_26312_MOESM1_ESM.docx]

**TITLE:** “**HIV and Vertebral Fractures: a Systematic Review and Metanalysis** ”

**Authors:** Thales ASH Ilha^1^, Fabio V Comim^1,2^, Rafaela M Copes^1,2^, Juliet E Compston^3^, and Melissa O Premaor^1,2 🖂^.

**Affiliations:**

1. Pós-graduação em Farmacologia, Health Sciences Center, Federal University of Santa Maria, Santa Maria (Brazil).

2. Department of Clinical Medicine, Health Sciences Center, Federal University of Santa Maria, Santa Maria (Brazil).

3. Cambridge Biomedical Campus, Cambridge (United Kingdom)

^🖂^ CORRESPONDING AUTHOR:

Melissa Orlandin Premaor, MD PhD

Departamento de Clínica Médica, Universidade Federal de Santa Maria (UFSM)

Sala 1337, Prédio 26 – CCS/ Avenida Roraima 1000, Campus UFSM

Santa Maria/ RS, Brazil.

Phone: 00 55 55 32208508; Fax: 00 55 55 32208018

E-mail: [premaor@ufsm.br](mailto:premaor@ufsm.br)

**SUPPLEMENTARY MATERIAL**

The terms used in this systematic review search include the Descriptors in Health Sciences (DeCS), MeSH and Emtree (EMBASE) terms, which were modified for each database. The terms used were: ("hiv" [MeSH Terms] OR "hiv" [All Fields]) AND ("spinal fractures" [MeSH Terms] OR ("spinal" [All Fields] AND "fractures" [All Fields]) OR "spinal fractures"[All Fields] OR ("vertebral"[All Fields] AND "fracture"[All Fields]) OR "vertebral fracture"[All Fields]) in Pubmed; (tw:(Fractures, Bone)) OR (tw:(Spinal Fracture)) AND (tw:( HIV)OR (tw:(Acquired Immunodeficiency Syndrome)OR (tw:(Anti-Retroviral Agents)), in Bireme; 1 'human immunodeficiency virus' OR 'antiretrovirus agent' OR 'acquired immunodeficiency' OR 'acquired immunodeficiency syndrome' AND 2 'spine fracture' AND 'fracture'/exp OR 'fracture' OR 'fragility fracture'/exp OR 'fragility fracture' OR 'vertebral fracture'/exp OR 'vertebral fracture' OR 'vertebral fracture assessment' AND 3 'human'/de AND ([adult]/lim OR [aged]/lim OR [middle aged]/lim OR [very elderly]/lim OR [young adult]/lim) in EMBASE; and (spine fracture:ti,ab,kw OR "fracture":ti,ab,kw) AND ("HIV positive":ti,ab,kw OR "AIDS":ti,ab,kw OR "antiretroviral therapies":ti,ab,kw), restricted only to clinical trial and cohort studies in COCHRANE.

| Table 2.Quality assessment of included studies. | | | | | |
| --- | --- | --- | --- | --- | --- |
|  |  | **Quality assessement criteria** | | | |
| **Author** | **Study design** | **Selection** | **Comparability** | **Outcome/ exposure** | **Overall quality** |
| Bedimo | Cohort | **** | * | * | 6 |
| Collin | Cohort | ** | - | * | 3 |
| Hansen | Cohort | *** | * | **** | 8 |
| Kurita | Cohort | ** | - | ** | 4 |
| Prieto-Alhambra | Case-control | *** | * | ** | 6 |
| Sharma | Cohort | ** | * | ** | 5 |
| Triant | Case-control | *** | * | - | 4 |
| Wormack | Cohort | *** | * | ** | 6 |
| Yang | Cohort | ** | - | ** | 4 |
| Yin (2012) | Cohort | *** | - | - | 3 |
| Yong | Case-control | ** | * | *** | 6 |
| Young | Cohort | * | * | ** | 4 |

Newcastle-Ottawa Scale was obtained to assess the selection, comparability and exposure of the case-control study, while the selection, comparability and outcome for the cohort study. -: no point; *: one point; **: two points; ***: three points; ****: four points.

| Table 3. Quality assessment, by Crombie’s items, of included cross-sectional studies | | | | | | | | |
| --- | --- | --- | --- | --- | --- | --- | --- | --- |
| **Studies** | **Q1** | **Q2** | **Q3** | **Q4** | **Q5** | **Q6** | **Q7** | **Total** |
| Borderi | Yes | Yes | Yes | Unclear | Yes | Yes | Yes | 6.5 |
| Ciullin | Yes | Yes | Yes | Unclear | Yes | Yes | Yes | 6.5 |
| Clò | No | Unclear | Yes | No | Unclear | Yes | Yes | 4 |
| Gazzola | Yes | Yes | Yes | Unclear | Yes | Yes | Yes | 6.5 |
| Mazzotta | No | Yes | Yes | Unclear | No | Yes | Yes | 4.5 |
| Pepe | Yes | Yes | Yes | Unclear | Yes | Yes | Yes | 6.5 |
| Porcelli | Yes | Yes | Yes | Unclear | Yes | Yes | Yes | 6.5 |
| Short | No | Yes | Yes | Unclear | No | Yes | Yes | 4.5 |
| Torti | Yes | Yes | Yes | Unclear | Unclear | Yes | Yes | 6 |
| Yin | Yes | Unclear | Yes | Unclear | Yes | Yes | Yes | 6 |

Q1 =Appropriateness of design to meet the aims; Q2 = Adequate description of the data; Q3= Report the response rates; Q4= Adequate representativeness of the sample to total; Q5= Clearly stated aims and likelihood of reliable and valid measurements; Q6 = Assessment of statistical significance; Q7= Adequate description of statistical methods. Yes = 1 point; Unclear = 0.5 points; No = 0 points.

Figure 4. Funnel plot of meta-analysis of the prevalence of vertebral fractures in HIV-positive subjects

Figure 5. Funnel plot of meta-analysis of the odds ratio of vertebral fractures in HIV-positive subjects
